# Life‐course of atopy and allergy‐related disease events in tropical sub‐Saharan Africa: A birth cohort study

**DOI:** 10.1111/pai.12719

**Published:** 2017-05-17

**Authors:** Swaib A. Lule, Harriet Mpairwe, Margaret Nampijja, Florence Akello, Joyce Kabagenyi, Benigna Namara, Gyaviira Nkurunungi, Dennison Kizito, Joseph Kahwa, Lawrence Muhangi, Stephen Nash, Moses Muwanga, Emily L. Webb, Alison M. Elliott

**Affiliations:** ^1^ London School of Hygiene and Tropical MedicineLondonUK; ^2^ MRC/UVRI Uganda Research UnitEntebbeUganda; ^3^ Entebbe HospitalEntebbeUganda

**Keywords:** birth cohort, wheeze, eczema, rhinitis, conjunctivitis, urticaria, atopy, Uganda, Africa

## Abstract

**Background:**

In high‐income countries, allergy‐related diseases (ARDs) follow a typical sequence, the ‘Atopic March’. Little is known about the life‐course of ARDs in the markedly different, low‐income, tropical environment. We describe ARDs in a tropical, African birth cohort.

**Methods:**

Ugandan children were followed from birth to 9 years. ISAAC questionnaires were completed at intervals; doctor‐diagnosed ARDs were recorded throughout follow‐up. Skin prick tests (SPTs) were performed at 3 and 9 years. Atopy was defined as ≥1 positive SPT.

**Results:**

Of the 2345 live‐born children, 1214 (52%) were seen at 9 years. Wheeze and eczema were common in infancy, but by 9 years, only 4% reported recent wheeze, 5% eczema and 5% rhinitis. Between 3 and 9 years, atopy prevalence increased from 19% to 25%. Atopy at 3 or 9 years was associated with reported ARD events at 9 years, for example OR = 5.2 (95% CI 2.9–10.7) for atopy and recent wheeze at 9 years. Reported or doctor‐diagnosed ARD events in early childhood were associated with the same events in later childhood, for example OR = 4.4 (2.3–8.4) for the association between reported wheeze before 3 years with reported recent wheeze at 9 years, but progression from early eczema to later rhinitis or asthma was not observed.

**Conclusion:**

Allergen sensitization started early in childhood and increased with age. Eczema and wheeze were common in infancy and declined with age. Atopy was strongly associated with ARD among the few affected children. The typical Atopic March did not occur. Environmental exposures during childhood may dissociate atopy and ARD.

Allergy‐related diseases (ARDs, such as asthma, eczema and rhinitis) affect approximately 20% of the world's population 1. Asthma affects nearly 300 million people 2 and eczema 15–30% of all children 3. Although ARDs have been uncommon in low‐ and middle‐income countries (LMIC), recent literature suggests they are increasing 4.

Atopy (defined by elevated allergen‐specific immunoglobulin E [asIgE] or skin prick test [SPT] positivity to common allergens) is associated with ARDs in high‐income countries (HICs) 5, but a heterogeneous picture is described in LMICs 5, 6, suggesting coexistence of atopic and non‐atopic ARD 7.

In HICs, eczema is usually the first presentation in the sequential manifestation of ARDs, and often lessens by age 4 years, when asthma and/or allergic rhinitis emerge, a transition known as the ‘Atopic [or Allergic] March’ 1. Early atopic sensitization is linked to the subsequent development of ARDs 8. However, little is known about the life‐course of ARDs in low‐income tropical environments, which differ from HICs in many aspects of lifestyle and the intensity of exposure to infections, especially chronic parasitic infections.

The Entebbe Mother and Baby Study (EMaBS) is a birth cohort, initially designed to investigate whether anthelminthic treatment in pregnancy and early childhood influences children's response to vaccines and infections 9. Helminths and anthelminthic treatment showed only modest effects on infants’ response to vaccines 10, 11, but hookworm infection in pregnancy was inversely associated with childhood eczema 12, and anthelminthic treatment in pregnancy increased the incidence of infantile 13 and childhood eczema 14. We describe the prevalence, phenotype, severity, inter‐relationships and life‐course of ARD events among children in this tropical birth cohort, to age 9 years.

## Methods

### Study population

The EMaBS is based in Entebbe municipality and Katabi subcounty, Wakiso district, Uganda: a peninsula on Lake Victoria, comprising urban, rural and fishing communities.

### Study design

Between 2003 and 2005, pregnant women were recruited into a randomized, double‐blind, placebo‐controlled trial of anthelminthic treatment in pregnancy and early childhood, and followed up as previously described [ISRCTN32849447] 9. The Research and Ethics Committee of the Uganda Virus Research Institute, the Uganda National Council for Science and Technology, and the London School of Hygiene and Tropical Medicine granted ethical approval.

### Investigation of allergy‐related disease outcomes

At ages 1, 2, 3, 5 and 9 years, caregivers were interviewed (in the child's presence) on ARD symptoms using questions from the International Study on Allergy and Asthma in Children (ISAAC) questionnaire 15 and data on urticaria were also collected. For data collected at age 3 years, these questions were included in the questionnaire from November 2007 onwards; hence, only responses from children attending their age 3 visit from that date onwards were collected.

*Reported recent events* were wheeze, eczema (a recurrent pruritic rash with typical infant or child distribution), allergic rhinitis (sneezing or runny nose or blocked nose, with itchy and watery eyes, without having a cold or ‘flu’) and urticaria (pruritic rash with wheals, ‘ebilogologo’ in the vernacular), occurring in the preceding 12 months.

At 9 years, the questionnaire was supplemented with (i) the ISAAC video questionnaire (VQ; shown after the oral questionnaire [OQ]), and (ii) questions from the UK diagnostic criteria (UKDC) for atopic eczema 15. The VQ contained five short sequences of asthma symptoms (audible wheezing without breathlessness, exercise‐induced wheezing, waking at night with breathlessness, nocturnal coughing, a severe asthma attack). Each sequence was followed by questions asking whether the child's breathing had ‘ever’ or ‘in the last 12 months’ been like the person's in the video. Children were examined for visible flexural dermatitis by team members trained in the standardized approach 16. The UKDC defines eczema as a recent pruritic rash with at least three of the following: history of flexural involvement, history of generally dry skin, personal history of asthma or allergic rhinitis, visible flexural dermatitis and onset below age 2 years.

*Doctor‐diagnosed ARD events* were identified when sick children presented to the study clinic. Wheezing episodes below the age of 5 years were documented. Asthma was diagnosed after the age of 5 years as an episode of wheezing or, a dry nocturnal cough, with a previous asthma‐like episode, after excluding other possible causes. Eczema was a recurrent pruritic rash lasting more than 6 months, with typical infant or child distributions.

### Atopic sensitization

Skin prick testing was performed in a subset of three‐year‐olds (those who attained 3 years of age from November 2007 onwards, when SPT was added to the procedures performed at this visit) and in nine‐year‐olds, on the volar surface of the arm using standard methods 17 with allergens likely to elicit a response in this setting 18. At 3 years 12, dust mites (*Dermatophagoides, Blomia tropicalis*), cow's milk and egg white were used; and at 9 years, the dust mites, German cockroach (*Blattella germanica*), peanut, Bermuda grass, cat, pollen and mould were used (ALK‐Abelló, Laboratory Specialities (Pty) Ltd, Randburg, South Africa). Wheals were measured after 15 min, positive being a mean diameter ≥3 mm.

The primary definition of atopy was SPT positivity to at least one allergen (further categorized as monosensitivity [sensitive to one allergen] and polysensitivity [≥2 allergens]).

At 9 years, plasma stored at −80°C was assessed for *Dermatophagoides‐*specific (Der‐p) IgE response using an in‐house ELISA as previously described 13. The lower detection limit was 312 ng/ml. A secondary definition of atopy was detectable Der‐p IgE >312 ng/ml.

### Statistical analysis

Data were double‐entered in Microsoft Access and analysed with Stata 14 (College Station, TX, USA). Chi‐square tests were used to compare maternal baseline (age, education, marital status, any worm infection, body mass index, anaemia) and child (sex, atopy at 3 years and infantile eczema diagnosis) characteristics between children seen and not seen at age 9 years. Agreement between OQ and VQ was determined by kappa statistic (κ) 19.

Cross‐sectional associations between atopy and reported recent ARD events at 9 years were estimated using logistic regression and population‐attributable fraction (PAF) calculated. Longitudinal inter‐relationships between atopy or reported ARDs in the first 3 years of life (reported ARD event at 1, 2 or 3 years) and atopy or reported ARDs at 9 years were examined using logistic regression. Poisson regression with random effects was used to assess whether reported ARDs or doctor‐diagnosed ARD events in early childhood (0–5 years) or atopy at 3 years were associated with doctor‐diagnosed ARD events between 5 and 9 years.

## Results

**Figure 1:**
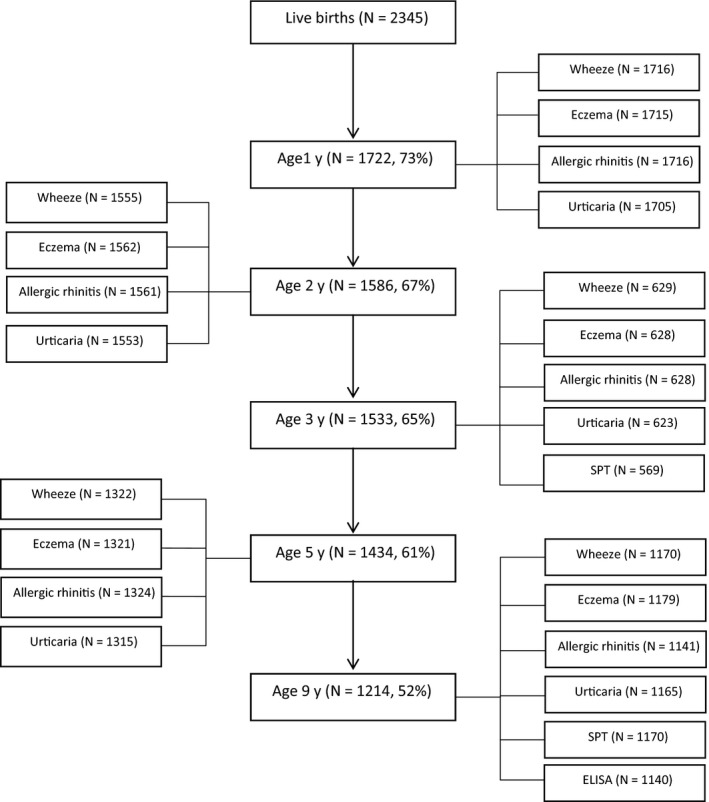
Flow chart showing number of the Entebbe Mother and Baby Study participants seen at each time point and those providing outcome information at each time point.

Of the 2345 live births, 1214 (52%) children were seen at 9 years. Fig. 1 shows the number of participants seen at each annual visit (1, 2, 3, 5 and 9 years) and the number for whom outcome data were collected at each time point. Of the nine‐year‐olds, 626 (52%) were males, 1203 (99%) underwent SPT, and 1140 (94%) had Der‐p IgE measured. Children seen at 9 years were similar to those not seen in terms of some maternal characteristics (marital status, BMI and worm infection at enrolment), child's sex, eczema diagnosis (before age 1 year) and atopy (3 years). On average, children seen at 9 years had older, better educated mothers from households with higher socio‐economic status at enrolment.

### Prevalence and life‐course of ARD outcomes and atopy

**Figure 2:**
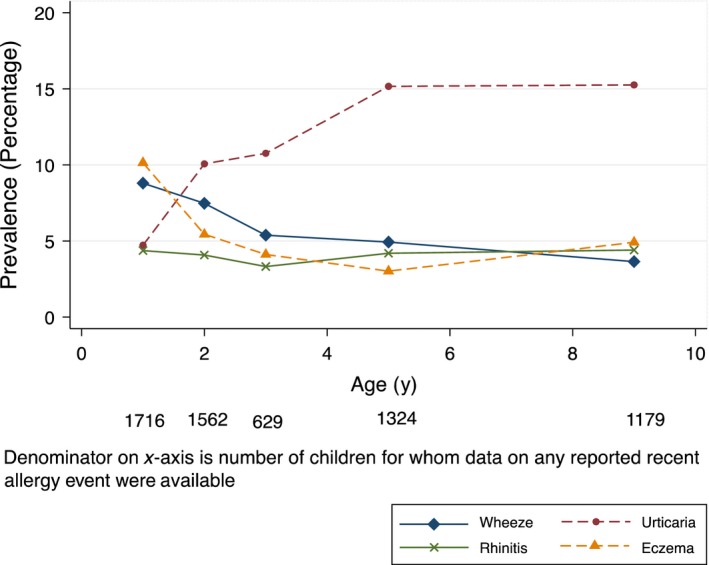
Proportion of children with reported recent allergy‐related events by age in the Entebbe Mother and Baby Study cohort. *Note:* missing data for given events at Year 1: eczema 1, urticaria 11; Year 2: wheeze 7, allergic rhinitis 1, urticaria 9; Year 3: eczema 1, allergic rhinitis 1, urticaria 6; Year 5: wheeze 2, eczema 3, urticaria 9; Year 9: wheeze 9, allergic rhinitis 38, urticaria 14.

Reported recent wheeze and eczema were most prevalent at 1 year and decreased with age. Reported recent urticaria increased and was the most prevalent reported event by 9 years. Allergic rhinitis was rare throughout childhood (Fig. 2).

At 9 years, by OQ, prevalence of recent wheeze was 3.8%, eczema 4.9%, allergic rhinitis 4.6% and urticaria 15.5% (Table 1). Of the 44 children with recent wheeze, 16 (36%) had suffered ≥4 attacks in the last 12 months, 12 (27%) reported speech interruption and 14 (32%) sleep disturbance. Fifteen children (1.3%) reported recent eczema based on the UKDC; of these 13 (87%) had their first episode before age 2 years. Of the 52 with allergic rhinitis, 9 (17%) reported interruptions in daily activities.

**Table 1:** Allergy‐related outcomes at 9 years of age in the Entebbe Mother and Baby Study birth cohort

Allergic symptoms	Present	Absent	Prevalence (95% CI)
*Oral questionnaire responses*			
Asthma symptoms			
Wheeze ever	75	1095	6.4% (5.1–8.0)
Reported recent wheeze	44	1126	3.8% (2.7–5.0)
Reported recent exercise‐induced wheeze	19	916	2.0% (1.2–3.2)
Reported recent dry cough at night	108	829	11.5% (9.6–13.7)
Eczema			
Reported recent eczema	58	1121	4.9% (3.8–6.3)
Reported recent eczema – UKDC	15	1161	1.3% (0.7–2.1)
Allergic rhinitis			
Allergic rhinitis ever	52	1089	4.6% (3.4–5.9)
Reported recent allergic rhinitis	52	1089	4.6% (3.4–5.9)
Urticaria			
Urticarial rash ever	261	912	22.3% (19.9–24.7)
Reported recent urticarial rash	180	985	15.5% (13.4–17.7)
*Video questionnaire responses*			
Wheeze			
Recent audible wheeze at rest	13	1161	1.1% (0.6–1.9)
Recent exercise‐induced wheeze	22	1150	1.9% (1.2–2.8)
Recent waking at night with wheeze	11	1159	0.9% (0.5–1.7)
Recent nocturnal coughing	54	1109	4.6% (3.5–6.0)
Recent wheeze with speech disturbance	14	1135	1.2% (0.7–2.0)
*Skin prick test reaction*			
Atopy (any single allergen)	292	867	25.2% (22.7–27.8)
Polysensitivity (≥2 positive allergens)	176	986	15.1% (13.1–17.3)
*Dermatophagoides mix*	210	958	18.0% (15.8–20.3)
*Blomia tropicalis*	174	989	15.0% (13.0–17.1)
German cockroach	128	1040	11.9% (9.2–12.9)
Peanut	16	1151	1.4% (0.8–2.2)
Bermuda Grass	14	1154	1.2% (0.7–2.0)
Cat	13	1151	1.1% (0.6–1.9)
Pollen	11	1155	0.9% (0.5–1.7)
Mould	3	1161	0.3% (0.1–0.8)
*IgE to Dermatophagoides pteronyssinus*			
Detectable level	335	805	29.4% (26.8–32.1)

Positive responses to the VQ were less common than to the OQ (Table 1). Comparing OQ and VQ, agreement was 97%, κ = 0.27, for recent wheeze vs. the scene showing audible wheezing without breathlessness: 98%, κ = 0.55, for exercise‐induced wheeze and 91%, κ = 0.40, for nocturnal coughing.

Between 5 and 9 years, rates of doctor‐diagnosed events were 8.2/1000 person‐years (pyr) for asthma, 10.8/1000 pyr for eczema and 9.3/1000 pyr for urticaria.

**Figure 3:**
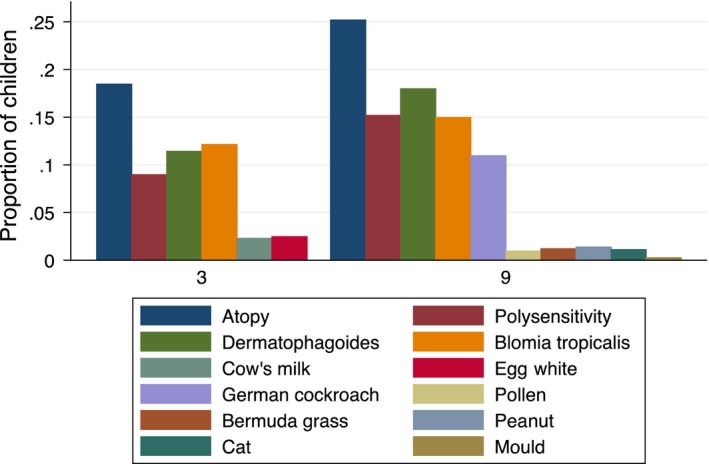
Trends in allergen sensitization, assessed by skin prick test response, by age (year) in the Entebbe Mother and Baby Study Cohort.

The commonest SPT responses were to dust mites at both 3 and 9 years (Table 1; Fig. 3). As previously described 12, 569 children underwent SPT at 3 years of age, of whom 105 (18%) were atopic, 11% positive for *Dermatophagoides* and 12% positive for *Blomia tropicalis*.

At 9 years, SPT data from 33 participants were excluded; three were excluded because saline (negative control) response was ≥3 mm and 30 were excluded because histamine (positive control) response was <3 mm. Thus, SPT results for at least one allergen were available from 1170 children. Overall, 25% of children were positive for at least one allergen, 18% positive for *Dermatophagoides*, 15% to *Blomia* and 12% to German cockroach. Polysensitivity was detected among 9% at 3 years and 15% at 9 years. Sensitivity to food allergens, pollen or mould was uncommon at three or 9 years (Fig. 3). There were 335 (29%) nine‐year‐olds with detectable Der‐p IgE.

### Associations between atopy and reported ARD events at 9 years

Skin prick test positivity was associated with all reported recent ARDs at 9 years (Table 2 (i)), with the strongest associations seen for wheeze and allergic rhinitis (PAF = 52.1% and 72.2%, respectively). Polysensitivity at 9 years was also associated with wheeze (OR = 7.4, 95% CI: 3.9–13.9), eczema (OR = 3.2, 95% CI: 1.8–5.7), allergic rhinitis (OR = 10.6, 95% CI: 5.8–19.2) and urticaria (OR = 2.6, 95% CI: 1.8–3.9). Atopy defined by detectable Der‐p IgE showed similar, but weaker, associations (Table 2 (ii)).

**Table 2:** Association between atopy and reported recent events among nine‐year‐old children in the Entebbe Mother and Baby Study birth cohort

Allergy‐related event	Atopy present N (%)	OR (95% CI)	PAF (95% CI)
*(i) Atopy defined by skin prick test response (at least one positive SPT)*			
Wheeze			
Absent	252/1066 (23.6)	1	
Present	26 /41 (63.4)	5.6 (2.9–10.7)	52.1% (41.7–57.5)
Eczema			
Absent	251/1057 (23.8)	1	
Present	29/58 (50.0)	3.2 (1.9–5.5)	34.4% (23.4–40.9)
Allergic rhinitis			
Absent	229/1028 (22.2)	1	
Present	40/51 (78.4)	12.7 (6.4–25.1)	72.2% (66.2–75.3)
Urticaria			
Absent	214/934 (22.9)	1	
Present	61/168 (36.3)	1.9 (1.4–2.7)	17.4% (9.5–23.0)
*(ii) Atopy defined by detectable allergen‐specific IgE to Dermatophagoides pteronyssinus*			
Wheeze			
Absent	297/1046 (28.4)	1	
Present	21/38 (55.3)	3.1 (1.6–6.0)	37.5% (21.2–46.0)
Eczema			
Absent	300/1038 (28.9)	1	
Present	22/55 (40.0)	1.6 (0.9–2.9)	15.6% (0.0–26.0)
Allergic rhinitis			
Absent	275/1008 (27.3)	1	
Present	32/49 (65.3)	5.0 (2.7–9.2)	52.3% (41.5–58.2)
Urticaria			
Absent	255/908 (28.1)	1	
Present	61/171 (35.7)	1.4 (1.0–2.0)	10.6% (0.2–17.9)

PAF, Population‐attributable fraction.

Missing values for atopy (SPT): wheeze 62; eczema 64; allergic rhinitis 62; urticaria 63.

Missing values for atopy (asIgE): wheeze 86; eczema 86; allergic rhinitis 84; urticaria 86.

Among reported recent events at 9 years, wheeze was associated with eczema (OR = 4.0, 95% CI: 1.7–9.4) and with allergic rhinitis (OR = 10.5, 95% CI: 5.0–22.0). Urticaria was associated more weakly with wheeze, eczema or allergic rhinitis (OR, 95% CI: 2.6 (1.3–5.1), 1.9 (1.0–3.5) and 2.1 (1.1–4.0), respectively).

### Longitudinal associations between outcomes at 3 and 9 years

Atopy at 3 years was strongly associated with atopy at 9 years and with reported ARD events (with the exception of urticaria) at 9 years (Table S1). Reported recent wheeze or eczema before 3 years was weakly associated with atopy at 9 years (Table S2). Children who reported a particular ARD event before 3 years were more likely to report the same recent ARD event at 9 years, again with the exception of urticaria. However, there was no evidence of association between reported eczema before age 3 years and any of the other reported ARD outcomes at 9 years (Table S1).

Regarding doctor‐diagnosed ARDs, children with a doctor‐diagnosed ARD before 5 years had higher rates of the same ARD between 5 and 9 years (Table S2), but doctor‐diagnosed eczema before 5 years was not associated with rates of other ARDs later in childhood (Table S2). Children with atopy at 3 years had higher rates of asthma in later childhood than those who were not atopic, but early atopy was not associated with later rates of eczema or urticaria. Associations between reported recent ARDs before 5 years and doctor‐diagnosed ARDs in later childhood showed similar patterns (Table S2).

## Discussion

This is the first description of the life‐course of allergy‐related conditions in tropical sub‐Saharan Africa. Eczema and wheeze were common in infancy, but prevalence declined markedly by 3 years, and there was no increase in later childhood. By contrast, atopic sensitization increased with age. However, atopy was strongly associated with eczema, wheeze and rhinitis, and these conditions were associated with each other. Atopy or ARD in early life was associated with later ARD, although the classic transition from eczema to rhinitis or asthma (the ‘Atopic March’) was not observed. Reported urticaria was an exception from this pattern: reported urticaria increased with age and showed only weak associations with atopy and other ARDs.

The strength of this study was the prospective collection of data on doctor‐diagnosed ARD throughout life, and of reported events and SPT sensitivity at key intervals. A concern was lack of familiarity among study participants and their families with terms such as wheeze, asthma, eczema and allergic rhinitis, which have no equivalents in the vernacular (Luganda).

Moderate agreement between the OQ and VQ at age nine was reassuring; the higher prevalence of wheeze by OQ may reflect the more severe presentations of asthma shown in the VQ 20. Reported wheeze is not synonymous with asthma, even among older children, but is regarded as the best tool for collection of epidemiological data on asthma 21. The children retained in the cohort at age 9 years, compared to those lost to follow‐up, came from relatively well‐to‐do families and as such are not fully representative of the original study population. Also, SPT at age 3 years was only performed on a (chronological) subset of cohort children due to study procedures being introduced when many children had already attended their three‐year annual visit. However, rates of infantile eczema and prevalence of atopy at 3 years were similar between those seen at 9 years and those seen as young children but later lost to follow‐up, and prevalence of ARDs at 9 years was similar among those who did and did not have SPT results at age 3 years.

Urticaria is a well‐recognized phenomenon, known in Luganda as ‘ebilogologo’. Given the differing life‐course of reported urticaria, and weak associations with atopy, we suspect that this represents a different phenomenon to other ARDs, possibly the long‐recognized response to active helminth infections 22 or exposure to insect venoms, increasing with age.

The prevalence of wheeze, eczema and rhinitis at 9 years in this population was low in relation to global findings from the ISAAC studies, which involved six‐ to seven‐year‐olds and 13‐ to 14‐year‐olds in many countries. Our results, compared to the ranges among younger and older participants at ISAAC centres, respectively, were as follows: for recent wheeze 4%, compared to 4–32% and 2–32% 23; for eczema 5% (1% using the more rigorous UK diagnostic criteria), compared to 2–16% and 1–17% 24; for rhinitis 5%, compared to 2–65% and 4–80% 25. In ISAAC populations, ARD prevalence tended to be lower in less affluent and more rural centres, and our findings accord with this pattern: EMaBS participants were drawn from a mixed urban–rural setting, recruited at the government hospital (an option usually chosen by less affluent women), and 85% of women were earning less than £10 per month at enrolment 26.

By contrast, we found a much stronger association between ARDs and atopy (measured by skin prick test) than we expected based on ISAAC. For wheeze, our point estimates for OR (5.2 [95% CI: 2.9–10.7]) and PAF (52%) exceeded the grouped estimate for ISAAC's affluent countries (4.0 [3.5–4.6] and 41%, respectively 5); similarly, for eczema, our OR and PAF estimates (3.2 [1.9–5.5] and 34%) exceeded ISAAC values for affluent settings (2.7 [2.3–3.1] and 28% 27). ISAAC's estimates for non‐affluent settings were about half the values for affluent countries, or lower. It is possible that these stronger associations could be partially explained by underreporting of ARDs in this setting, where directly translatable terms for asthma, eczema and rhinitis are not available.

The concept of the ‘Atopic March’ suggests that an impaired skin barrier (perhaps related to mutations in, for example, the filaggrin gene) renders individuals susceptible to atopic sensitization and thereafter vulnerable to atopic eczema and later to atopic rhinitis and atopic asthma 1. Our data show that atopic sensitization was acquired early and increased with age among children in this population, to levels comparable to affluent settings 28 and that early sensitization was associated with later ARD. However, despite these findings, the vast majority of children with early atopic sensitization, eczema or wheeze had no evidence of ARD by age 9 years.

We, and others, have previously shown the importance of early life (including prenatal) exposures in ARD risk 12, 29. However, these new data suggest that environmental factors experienced later in life can further dissociate acquired atopy from ARD risk, thus preventing the Atopic March. Possible candidates include immunomodulating infections such as helminths and malaria, which are common in this setting. A separate analysis of risk factors for ARD in this cohort may shed further light on the role of particular exposures, including infections, in this process. Tropical populations may be at high risk for atopic ARD if and when such protective exposures are removed.

## Funding

Wellcome Trust grants 064693, 079110, 95778; additional support from the UK Medical Research Council (MRC) and UK Department for International Development (DfID) under the MRC/DfID concordat.

## Supporting information


**Table S1.** Longitudinal associations between reported ARD events or atopy (SPT) in the first three years of life and reported ART events or atopy at age nine years among children in the Entebbe Mother and Baby Study birth cohort.
**Table S2.** Association between events (doctor diagnosed or reported recent) early in life or atopy early in life, and doctor‐diagnosed events between age five and nine years among children in the Entebbe Mother and Baby Study birth cohort.
