# Supplementary material for: Life‐course of atopy and allergy‐related disease events in tropical sub‐Saharan Africa: A birth cohort study
Source: Pediatr Allergy Immunol. 2017 May 17;28(4):377–83. doi: 10.1111/pai.12719 (PMC5488189; doi:10.1111/pai.12719)
Supplement: Supplementary file 1 — Table S1. Longitudinal associations between reported ARD events or atopy (SPT) in the first three years of life and reported ART events or atopy at age nine years among children in the Entebbe Mother and Baby Study birth cohort. Table S2. Association between events (doctor diagnosed or reported recent) early in life or atopy early in life, and doctor‐diagnosed events between age five and nine years among children in the Entebbe Mother and Baby Study birth cohort. [file PAI-28-377-s001.docx]

| **Supplementary table 1** | | | | | **Longitudinal associations between reported ARD events or atopy (SPT) in the first three years of life and reported ART events or atopy at age nine years among children in the Entebbe Mother and Baby Study birth cohort** | | | | | | | | | | | | | | | | | | | | | | | | | | |  |
| --- | --- | --- | --- | --- | --- | --- | --- | --- | --- | --- | --- | --- | --- | --- | --- | --- | --- | --- | --- | --- | --- | --- | --- | --- | --- | --- | --- | --- | --- | --- | --- | --- |
| **Reported recent ARD between age 0-3 years** | | | **Reported recent allergy-related events and atopy at age nine years** | | | | | | | | | | | | | | | | | | | | | | | | | | | | |  |
|  |  |  | **Wheeze present** | | |  | **Eczema present** | | | | | |  | | **Allergic rhinitis present** | | | | |  | | **Urticaria present** | | |  | | **SPT^1^ at 9 years**  **Present** | | | | |  |
|  | | **N (%)** | | **OR (95% CI)** | |  | | **N (%)** | **OR 95% (CI)** | | |  | | **N (%)** | | **OR 95% (CI)** | | |  | | **N (%)** | | **OR 95% (CI)** | | |  | | **N (%)** | **OR 95% (CI)** | | |  |
| **Wheeze** | | | |  | |  | | | |  | | | |  | | | |  | | | | | |  | |  | |  | |  |  |  |
| Absent | 25/949 (3) | | | 1 | | 44/958 (5) | | | | | 1 | | | 40/931 (4) | | | 1 | | | | 147/947 (16) | | | 1 | | | | 187/944 (20) | | 1 | |  |
| Present | 17/159 (10) | | | **4.4 (2.3, 8.4)** | | 11/159 (7) | | | | | 1.5 (0.8, 3.0) | | | 11/151 (7) | | | 1.8 (0.9, 3.5) | | | | 22/156 (14) | | | 0.9 (0.6, 1.4) | | | | 40/151 (26) | | **1.5 (1.0, 2.2)** | |  |
| Eczema | | | |  | |  | | | | |  | | |  | | |  | | | |  | | |  | | | |  | |  | |  |
| Absent | 36/958 (4) | | | 1 | | 39/966 (4) | | | | | 1 | | | 41/934 (4) | | | 1 | | | | 140/953 (15) | | | 1 | | | | 188/949 (20) | | 1 | |  |
| Present | 6/150 (4) | | | 1.1 (0.4, 2.6) | | 16/151 (11) | | | | | **2.8 (1.5, 5.2)** | | | 10/148 (7) | | | 1.6 (0.8, 3.2) | | | | 29 /150 (19) | | | 1.4 (0.9, 2.2) | | | | 39/146 (27) | | **1.5 (1.0, 2.2)** | |  |
| Allergic rhinitis | | | |  | |  | | | | |  | | |  | | |  | | | |  | | |  | | | |  | |  | |  |
| Absent | 38/1019 (4) | | | 1 | | 46/1028 (4) | | | | | 1 | | | 42/995 (4) | | | 1 | | | | 157/1015 (15) | | | 1 | | | | 208/1004 (21) | | 1 | |  |
| Present | 4/89 (5) | | | 1.2 (0.4, 3.5) | | 9/89 (10) | | | | | **2.4 (1.1, 5.1)** | | | 9/87 (10) | | | **2.6 (1.2, 5.6)** | | | | 12/88 (14) | | | 0.9 (0.5, 1.6) | | | | 19/91 (21) | | 1.0 (0.6, 1.7) | |  |
| Urticaria |  | | |  | |  | | | | |  | | |  | | |  | | | |  | | |  | | | |  | |  | |  |
| Absent | 32/948 (3) | | | 1 | | 40/957 (4) | | | | | 1 | | | 41/928 (4) | | | 1 | | | | 140/945 (15) | | | 1 | | | | 188/935 (20) | | 1 | |  |
| Present | 10/160 (6) | | | 1.9 (0.9, 4.0) | | 15/160 (9) | | | | | **2.4 (1.3, 4.4)** | | | 10/154 (6) | | | 1.5 (0.7, 3.1) | | | | 29/158 (18) | | | 1.3 (0.8, 2.0) | | | | 39/160 (24) | | 1.3 (0.9, 1.9) | |  |
| Atopy at 3 years | | | |  | |  | | | | |  | | |  | | |  | | | |  | | |  | | | |  | |  | |  |
| Negative | 7/296(2) | | | 1 | | 8/299 (3) | | | | | 1 | | | 6/294 (2) | | | 1 | | | | 35/296 (12) | | | 1 | | | | 34/290 (12) | | 1 | |  |
| Positive | 9/67 (13) | | | **6.4 (2.3, 17.9)** | | 11/67 (16) | | | | | **7.1 (2.8, 18.6)** | | | 10/64 (16) | | | **8.9 (3.1, 25.5)** | | | | 12/66 (18) | | | 1.7 (0.8, 3.4) | | | | 36/66 (55) | | **9.0 (4.9, 16.5)** | |  |
| Mono-sensitivity *Dermatophagoides* at 3 years | | | | | |  | | | | |  | | |  | | |  | | | |  | | |  | | | |  | |  | |  |
| Negative | 13/353 (4) | | | 1 | | 17/356 (5) | | | | | 1 | | | 12/348 (3) | | | 1 | | | | 44/352 (13) | | | 1 | | | | 64/347 (18) | | 1 | |  |
| Positive | 3/10 (30) | | | **11.2 (2.6, 48.3)** | | 2/10 (20) | | | | | 5.0 (1.0, 25.3) | | | 4/10 (40) | | | **18.7 (4.6, 74.9**) | | | | 3/10 (30) | | | 2.1 (0.7, 12.0) | | | | 6/9 (67) | | **8.8 (2.2, 36,3)** | |  |
| Mono-sensitivity *Blomia tropicalis* at 3 years | | | | | |  | | | | |  | | |  | | |  | | | |  | | |  | | | |  | |  | |  |
| Negative | 16/354 (5) | | | 1 | | 18/357 (5) | | | | | 1 | | | 15/349 (4) | | | 1 | | | | 47/354 (13) | | | 1 | | | | 65/347 (19) | | 1 | |  |
| Positive | 0/9 (0) | | | - | | 1/9 (11) | | | | | 2.4 (0.3, 19.9) | | | 1/9 (11) | | | 2.8 (0.3, 23.7) | | | | 0/8 (0) | | | - | | | | 5/9 (56) | | **5.4 (1.5, 20.8)** | |  |
| Mono-sensitivity cow’s milk at 3 years | | | | | |  | | | | |  | | |  | | |  | | | |  | | |  | | | |  | |  | |  |
| Negative | 16/357 (5) | | | 1 | | 19/360 (5) | | | | | 1 | | | 16/352 (5) | | | 1 | | | | 45/356 (12.6) | | | 1 | | | | 69/350 (20) | | 1 | |  |
| Positive | 0/6 (0) | | | - | | 0/6 (0) | | | | | - | | | 0/6 (0) | | | - | | | | 2/6 (33) | | | 3.5 (0.6, 19.4) | | | | 1/6 (17) | | 0.8 (0.1, 7.1) | |  |
| Mono-sensitivity egg white at 3 years | | | | | |  | | | | |  | | |  | | |  | | | |  | | |  | | | |  | |  | |  |
| Negative | 16/355 (4.5) | | | 1 | | 17/358 (5) | | | | | 1 | | | 16/350 (5) | | | 1 | | | | 45/354 | | | 1 | | | | 67/348 (19) | | 1 | |  |
| Positive | 0/8 (0) | | | **-** | | 2/8 (25) | | | | | **6.7 (1.3, 35.6)** | | | 0/8 (0.0) | | | - | | | | 2/8 (25) | | | 2.3 (0.4, 11.6) | | | | 3/8 (38) | | 2.5 (0.6, 10.8) | |  |
| ^1^ SPT: Skin prick test positivity to *Dermatophagoides* or *Blomia tropicalis*  OR: Odds Ratio, CI: confidence interval,  Associations with P<0.05 are highlighted in **bold** | | | | | | | | | | | | | | | | | | | | | | | | | | | | | | | |  |

| **Supplementary table 2** | | | | **Association between events (doctor diagnosed or reported recent) early in life or atopy early in life, and doctor-diagnosed events between age five and nine years among children in the Entebbe Mother and Baby Study birth cohort** | | | | | | | | | | |
| --- | --- | --- | --- | --- | --- | --- | --- | --- | --- | --- | --- | --- | --- | --- |
|  | |  | **Doctor-diagnosed events age 5-9 years** | | | | | | | | | | | |
|  |  |  | **Asthma** | | | |  | **Eczema** | | |  | **Urticaria** | | |
|  |  | **Pyrs (x1000)** | **Events** | | **Rate per 1000 pyrs** | **RR (95% CI)** |  | **Events** | **Rate per 1000 pyrs** | **RR (95% CI)** |  | **Events** | **Rate per 1000 pyrs** | **RR (95% CI)** |
| **Doctor-diagnosed ARD events (0-5 years)** | | | | | | |  |  |  |  |  |  |  |  |
| Asthma-like illness | | | | |  |  | |  |  |  | |  |  |  |
|  | Absent | 5.92 | 28 | | 4.7 | 1 | | 64 | 10.8 | 1 | | 56 | 9.5 | 1 |
|  | Present | 0.18 | 22 | | 121.6 | **28.0 (2.5, 311.1)** | | 2 | 11.0 | 1.0 (0.1, 9.4) | | 1 | 5.5 | 0.6 (0.1, 4.5) |
| Eczema | |  |  | |  |  | |  |  |  | |  |  |  |
|  | Absent | 5.26 | 36 | | 6.8 | 1 | | 19 | 3.6 | 1 | | 51 | 9.7 | 1 |
|  | Present | 0.84 | 14 | | 16.7 | 2.7 (0.5, 14.5) | | 47 | 56.3 | **15.9 (7.6, 33.4)** | | 6 | 7.2 | 0.7 (0.3, 1.8) |
| Urticaria | |  |  | |  |  | |  |  |  | |  |  |  |
|  | Absent | 5.47 | 47 | | 8.6 | 1 | | 62 | 11.3 | 1 | | 45 | 8.2 | 1 |
|  | Present | 0.63 | 3 | | 4.8 | 0.5 (0.0, 4.6) | | 4 | 6.4 | 0.5 (0.1, 2.3) | | 12 | 19.1 | **2.3 (1.2, 4.6)** |
| **Reported recent ARD events (0-5 years)** | | | | | | | |  |  |  | |  |  |  |
| Wheeze | |  |  | |  |  | |  |  |  | |  |  |  |
|  | No | 4.99 | 7 | | 1.4 | 1 | | 38 | 7.6 | 1 | | 48 | 9.6 | 1 |
|  | Yes | 0.9 | 37 | | 40.1 | **28.7 (8.8, 93.6)** | | 27 | 29.2 | 3.4 (1.0, 11.6) | | 9 | 9.7 | 1.0 (0.5, 2.1) |
| Eczema | |  |  | |  |  | |  |  |  | |  |  |  |
|  | No | 5.03 | 39 | | 7.8 | 1 | | 26 | 5.2 | **1** | | 44 | 8.7 | 1 |
|  | Yes | 0.88 | 5 | | 5.7 | 0.7 (0.1, 4.5) | | 39 | 44.4 | **8.7 (3.9, 19.4)** | | 13 | 14.8 | 1.7 (0.9, 3.3) |
| Allergic rhinitis | | | | |  |  | |  |  |  | |  |  |  |
|  | No | 5.31 | 21 | | 4.0 | **1** | | 56 | 10.5 | 1 | | 50 | 9.4 | 1 |
|  | Yes | 0.60 | 23 | | 38.3 | **9.2 (1.8, 47.9)** | | 9 | 15.0 | 1.4 (0.4, 4.9) | | 7 | 11.6 | 1.2 (0.5, 2.9) |
| Urticaria | |  |  | |  |  | |  |  |  | |  |  |  |
|  | No | 4.54 | 33 | | 7.3 | 1 | | 45 | 9.9 | 1 | | 40 | 8.8 | 1 |
|  | Yes | 1.37 | 11 | | 8.0 | 1.0 (0.2, 4.8) | | 20 | 14.6 | 1.4 (0.6, 3.4) | | 17 | 12.4 | 1.4 (0.8, 2.6) |
| **Atopy at three years** | | |  | |  |  | |  |  |  | |  |  |  |
|  | No | 1.50 | 3 | | 2.0 | 1 | | 14 | 9.3 | 1 | | 20 | 13.3 | 1 |
|  | Yes | 0.32 | 17 | | 52.4 | **25.0 (3.6, 171.9)** | | 1 | 3.1 | 0.3 (0.0, 3.3) | | 3 | 9.2 | 0.7 (0.2, 2.5) |
| Pyrs: person years at risk, CI: confidence interval, RR: Rate ratio  Associations with P<0.05 are highlighted in **bold** | | | | | | | | | | | | | | |
